# The mean value theorem and Taylor’s theorem for fractional derivatives with Mittag–Leffler kernel

**DOI:** 10.1186/s13662-018-1543-9

**Published:** 2018-03-09

**Authors:** Arran Fernandez, Dumitru Baleanu

**Affiliations:** 1 Department of Applied Mathematics and Theoretical Physics, University of Cambridge, Cambridge, UK; 2 Department of Mathematics, Cankaya University, Ankara, Turkey; 3 Institute of Space Sciences, Magurele-Bucharest, Romania

**Keywords:** Fractional calculus, Mean value theorem, Taylor’s theorem, Mittag–Leffler kernel

## Abstract

We establish analogues of the mean value theorem and Taylor’s theorem for fractional differential operators defined using a Mittag–Leffler kernel. We formulate a new model for the fractional Boussinesq equation by using this new Taylor series expansion.

## Introduction

The importance of fractional calculus, i.e. the study of differentiation and integration to non-integer orders, started to be appreciated during the last few decades, mainly because many successful models were developed in various branches of science and engineering. There are several different definitions for derivatives and integrals (together referred to as *differintegrals*) in the fractional sense, which are classified in different categories. For example, the classical *Riemann–Liouville* and *Caputo* formulae are defined by integral transforms with power function kernels [1–4], while some more recent formulae [5–9] use integral transforms with various other kernel functions.

Fractional derivatives and integrals have found many applications across a huge variety of fields of science—for example in financial models [10], geohydrology [11], chaotic systems [12], epidemiology [13–15], drug release kinetics [16–19], nuclear dynamics [20], viscoelasticity [21], complexity theory [22], bioengineering [23], image processing [24], and so on. One of the reasons for their broad usefulness is their *non-locality*: ordinary derivatives are local operators, while fractional ones (at least according to most definitions) are non-local, having some degree of memory. For this reason, they are often useful in problems involving global optimisation, such as those appearing in control theory.

Fractional calculus is one of the most swiftly growing areas in mathematics, and during recent years, researchers have been trying to use it in the treatment of dynamics of complex systems [22, 25]. Some of these have complicated dynamics which cannot be described properly with classical fractional models, and therefore it has been necessary to develop new fractional operators. In this paper, we shall consider fractional calculus according to a relatively new definition [7], usually referred to as the AB formula, which has a stronger connection to the non-locality properties of fractional calculus. In this model, the fractional integral operator is defined by

$${}^{\mathrm{AB}}_{}I^{\alpha}_{a+}\, f(t)= \frac{1-\alpha}{B(\alpha)}f(t)+\frac {\alpha}{B(\alpha)}{}^{\mathrm{RL}}_{}I_{a+}^{\alpha}\, f(t), $$

while the fractional differential operator can be defined in two different ways, labelled ABR and ABC for Riemann–Liouville type and Caputo type:

$$\begin{aligned}& {}^{ABR}_{}D^{\alpha}_{a+}\, f(t)=\frac{B(\alpha)}{1-\alpha} \frac {\mathrm{d}}{\mathrm{d}t} \int_{a}^{t}f(x)E_{\alpha}\biggl( \frac{-\alpha }{1-\alpha}(t-x)^{\alpha} \biggr)\, \mathrm{d}x; \\& {}^{\mathrm{ABC}}_{}D^{\alpha}_{a+}\, f(t)= \frac{B(\alpha)}{1-\alpha} \int _{a}^{t}f'(x)E_{\alpha}\biggl(\frac{-\alpha}{1-\alpha}(t-x)^{\alpha} \biggr) \, \mathrm{d}x. \end{aligned}$$

In each case the functions and variables used satisfy the following requirements [26]: $a< t< b$ in $\mathbb{R}$; $\alpha\in(0,1)$; $B(\alpha)$ is a normalisation function satisfying $B>0$ and $B(0)=B(1)=1$; and $f:[a,b]\rightarrow\mathbb{R}$ is an $L^{1}$ function or, in the case of the ABC derivative, a differentiable function with $f'\in L^{1}$.

Certain fundamental results of calculus have already been established in the AB model: Laplace transforms [7], integration by parts [27], the product rule and chain rule [26], etc. But as the idea is still so new, much remains to be done in this area. Furthermore, the AB model has found various applications, for example in chaos theory [28], variational calculus [27], and oscillators [29].

Specifically, our aim is to prove generalised versions of the mean value theorem and Taylor’s theorem in the AB model of fractional calculus. Analogous results are already known in the standard Riemann–Liouville [30] and Caputo [31] models, and versions of the mean value theorem for fractional *difference* operators have been proved in both the Caputo–Fabrizio model [32] and the AB model [33], but a fractional mean value theorem in the continuous AB model has not been established up until now. We shall also demonstrate some real-world applications of our results for modelling problems in fluid dynamics using a new fractional Boussinesq equation.

Our paper is structured as follows. In Sect. 2 we prove the main results and all required lemmas, and in Sect. 3 we redconsider some example Taylor expansions and discuss potential applications of our results.

## Main results

### The mean value theorem

The following result has been proved for example in [34], using Laplace transforms, and also in [26] using only the definition of AB derivatives and integrals.

#### Theorem 2.1

(AB Newton–Leibniz theorem)

*AB integrals and derivatives of Caputo type satisfy the following inversion relation*:

1 $$ {}^{\mathrm{AB}}_{}I_{a+}^{\alpha} {}^{\mathrm{ABC}}_{}D_{a+}^{\alpha}\, f(t)=f(t)-f(a) $$

*for* $0<\alpha<1$, $a< t< b$ *in* $\mathbb{R}$, *and* $f:[a,b]\rightarrow \mathbb{R}$ *differentiable such that* $f'$ *and* ${}^{\mathrm{ABC}}_{}D_{a+}^{\alpha}\, f$ *are both in* $L^{1}[a,b]$.

We can use this fact to prove the following analogue of the mean value theorem for fractional derivatives in the AB model.

#### Theorem 2.2

(AB mean value theorem)

*Let* $0<\alpha<1$, $a< b$ *in* $\mathbb{R}$, *and* $f:[a,b]\rightarrow\mathbb {R}$ *differentiable such that* $f'\in L^{1}[a,b]$ *and* ${}^{\mathrm{ABC}}_{}D_{a+}^{\alpha}\, f\in C[a,b]$. *Then*, *for any* $t\in[a,b]$, *there exists* $\xi\in[a,t]$ *such that*

2 $$ f(t)=f(a)+\frac{1-\alpha}{B(\alpha)}{}^{\mathrm{ABC}}_{}D_{a+}^{\alpha }\, f(t)+\frac{(t-a)^{\alpha}}{B(\alpha)\Gamma(\alpha)}{}^{ \mathrm{ABC}}_{}D_{a+}^{\alpha}\, f(\xi). $$

#### Proof

By Theorem 2.1, we have

$$\begin{aligned} f(t)-f(a)&={}^{\mathrm{AB}}_{}I_{a+}^{\alpha} \bigl( {}^{\mathrm{ABC}}_{}D_{a+}^{\alpha}\, f(t) \bigr) \\ &=\frac{1-\alpha}{B(\alpha)}{}^{\mathrm{ABC}}_{}D_{a+}^{\alpha}\, f(t)+\frac {\alpha}{B(\alpha)}{}^{\mathrm{RL}}_{}I_{a+}^{\alpha} \bigl({}^{\mathrm{ABC}}_{}D_{a+}^{\alpha}\, f(t) \bigr) \\ &=\frac{1-\alpha}{B(\alpha)}{}^{\mathrm{ABC}}_{}D_{a+}^{\alpha}\, f(t)+\frac {\alpha}{B(\alpha)\Gamma(\alpha)} \int_{a}^{t}(t-x)^{\alpha-1} \bigl({}^{ \mathrm{ABC}}_{}D_{a+}^{\alpha}\, f(x) \bigr)\,\mathrm{d}x. \end{aligned}$$

Now, by the integral mean value theorem, since ${}^{\mathrm{ABC}}_{}D_{a+}^{\alpha}\,f(x)$ is continuous and $(t-x)^{\alpha-1}$ is integrable and positive, there exists $\xi\in(a,t)$ such that

$$\begin{aligned} f(t)-f(a)&=\frac{1-\alpha}{B(\alpha)}{}^{\mathrm{ABC}}_{}D_{a+}^{\alpha } \,f(t)+\frac{\alpha}{B(\alpha)\Gamma(\alpha)}{}^{\mathrm{ABC}}_{}D_{a+}^{\alpha} \,f(\xi) \int_{a}^{t}(t-x)^{\alpha-1}\,\mathrm{d}x \\ &=\frac{1-\alpha}{B(\alpha)}{}^{\mathrm{ABC}}_{}D_{a+}^{\alpha} \,f(t)+\frac {\alpha}{B(\alpha)\Gamma(\alpha)}{}^{\mathrm{ABC}}_{}D_{a+}^{\alpha} \,f(\xi )\frac{(t-a)^{\alpha}}{\alpha}, \end{aligned}$$

as required. □

For interest’s sake we also include the following corollary, another form of the ABC fractional mean value theorem in terms of an inequality.

#### Corollary 2.1

*With all notations and assumptions as in Theorem *2.2, *if* *f* *is monotonic* (*increasing or decreasing*), *then*

3 $$ f(t)\geq f(a)+ \biggl[1+E_{\alpha} \biggl( \frac{-\alpha}{1-\alpha }(t-a)^{\alpha} \biggr) \biggr]^{-1} \frac{(t-a)^{\alpha}}{B(\alpha)\Gamma (\alpha)}{}^{\mathrm{ABC}}_{}D_{a+}^{\alpha}\,f( \xi) $$

*for some* $\xi\in(a,t)$.

#### Proof

We shall start from equation (2) to derive this inequality. Firstly, using the integral mean value theorem again, we can write the ABC derivative as

$$\begin{aligned} \frac{1-\alpha}{B(\alpha)}{}^{\mathrm{ABC}}_{}D_{a+}^{\alpha} \,f(t)&= \int _{a}^{t}f'(x)E_{\alpha}\biggl(\frac{-\alpha}{1-\alpha}(t-x)^{\alpha} \biggr)\,\mathrm{d}x \\ &=E_{\alpha} \biggl(\frac{-\alpha}{1-\alpha}(t-c)^{\alpha} \biggr) \int _{a}^{t}f'(x)\,\mathrm{d}x \\ &= \bigl(f(t)-f(a) \bigr)E_{\alpha} \biggl(\frac{-\alpha}{1-\alpha }(t-c)^{\alpha} \biggr) \end{aligned}$$

for some $c\in(a,t)$, since $E_{\alpha}$ is continuous and $f'$ is integrable and has constant sign. We substitute this into (2) to find

$$f(t)-f(a)= \bigl(f(t)-f(a) \bigr)E_{\alpha} \biggl(\frac{-\alpha}{1-\alpha }(t-c)^{\alpha} \biggr)+\frac{(t-a)^{\alpha}}{B(\alpha)\Gamma(\alpha )}{}^{\mathrm{ABC}}_{}D_{a+}^{\alpha} \,f(\xi), $$

and therefore

$$f(t)=f(a)+ \biggl[1-E_{\alpha} \biggl(\frac{-\alpha}{1-\alpha}(t-c)^{\alpha } \biggr) \biggr]^{-1}\frac{(t-a)^{\alpha}}{B(\alpha)\Gamma(\alpha )}{}^{\mathrm{ABC}}_{}D_{a+}^{\alpha}\,f(\xi). $$

Since the Mittag–Leffler function on a negative argument is completely monotone [35], the result follows. □

### Taylor’s theorem

Before starting to prove analogues of Taylor’s theorem for fractional AB derivatives, we first establish the following lemma.

#### Lemma 2.1

*If* $\alpha\in(0,1)$ *and* $a< b$ *in* $\mathbb{R}$ *and* $f:[a,b]\rightarrow \mathbb{R}$ *is a differentiable function such that* $f'$ *and all functions of the form* $({}^{\mathrm{ABC}}_{}D_{a+}^{\alpha} )^{m}f(t)$, $m\in\mathbb{N}$, *are* $L^{1}$ *functions*, *then*

4 $$\begin{aligned}& \bigl({}^{\mathrm{AB}}_{}I_{a+}^{\alpha} \bigr)^{m} \bigl({}^{\mathrm{ABC}}_{}D_{a+}^{\alpha} \bigr)^{m}f(t)- \bigl({}^{\mathrm{AB}}_{}I_{a+}^{\alpha } \bigr)^{m+1} \bigl({}^{\mathrm{ABC}}_{}D_{a+}^{\alpha} \bigr)^{m+1}f(t) \\& \quad =\frac{ ({}^{\mathrm{ABC}}_{}D_{a+}^{\alpha} )^{m}f(a)}{B(\alpha )^{m}}\sum_{k=0}^{m} \frac{\binom{m}{k}(1-\alpha)^{m-k}\alpha^{k}}{\Gamma (k\alpha+1)}(t-a)^{k\alpha} \end{aligned}$$

*for all* $m\in\mathbb{N}$.

#### Proof

By Theorem 2.1, we know that

5 $$ \bigl(1-{}^{\mathrm{AB}}_{}I_{a+}^{\alpha} {}^{\mathrm{ABC}}_{}D_{a+}^{\alpha } \bigr)f(t)=f(a). $$

So the left-hand side of equation (4) can be written as follows, where we denote ${}^{\mathrm{AB}}_{}I_{a+}^{\alpha}$ and ${}^{\mathrm{ABC}}_{}D_{a+}^{\alpha}$ by simply $I^{\alpha}$ and $D^{\alpha }$, respectively, for ease of notation:

$$\begin{aligned} & \bigl(I^{\alpha} \bigr)^{m} \bigl(D^{\alpha} \bigr)^{m}f(t)- \bigl(I^{\alpha} \bigr)^{m+1} \bigl(D^{\alpha} \bigr)^{m+1}f(t) \\ &\quad = \bigl(I^{\alpha} \bigr)^{m} \bigl(D^{\alpha} \bigr)^{m}f(t)- \bigl(I^{\alpha} \bigr)^{m} \bigl(I^{\alpha}D^{\alpha} \bigr) \bigl(D^{\alpha} \bigr)^{m}f(t) \\ &\quad = \bigl(I^{\alpha} \bigr)^{m} \bigl(1-I^{\alpha}D^{\alpha} \bigr) \bigl(D^{\alpha} \bigr)^{m}f(t) = \bigl(I^{\alpha} \bigr)^{m} \bigl( \bigl(D^{\alpha} \bigr)^{m}f(a) \bigr), \end{aligned}$$

where for the last step we used identity (5). Denoting the constant $(D^{\alpha} )^{m}f(a)$ by *A*, we have

$$\begin{aligned} \begin{aligned} \bigl({}^{\mathrm{AB}}_{}I_{a+}^{\alpha} \bigr)^{m}(A)&= \biggl(\frac{1-\alpha }{B(\alpha)}+\frac{\alpha}{B(\alpha)}{}^{\mathrm{RL}}_{}I_{a+}^{\alpha } \biggr)^{m}(A) \\ &=\frac{1}{B(\alpha)^{m}}\sum_{k=0}^{m} \binom{m}{k}(1-\alpha)^{m-k}\alpha ^{k}{}^{\mathrm{RL}}_{}I_{a+}^{k\alpha}(A) \\ &=\frac{A}{B(\alpha)^{m}}\sum_{k=0}^{m} \binom{m}{k}(1-\alpha)^{m-k}\alpha ^{k}\frac{(t-a)^{k\alpha}}{\Gamma(k\alpha+1)}, \end{aligned} \end{aligned}$$

as required. □

Now we are finally in a position to prove the following main result, our first analogue of Taylor’s theorem for fractional derivatives in the ABC model.

#### Theorem 2.3

(AB Taylor series about $t=a$)

*If* $\alpha\in(0,1)$ *and* $n\in\mathbb{N}$ *and* $a< b$ *in* $\mathbb{R}$ *and* $f:[a,b]\rightarrow\mathbb{R}$ *is a differentiable function such that* $f'$ *and all functions of the form* $({}^{\mathrm{ABC}}_{}D_{a+}^{\alpha} )^{m}f(t)$, $m\in\mathbb{N}$, *are* $L^{1}$ *functions*, *then for all* $t\in[a,b]$,

6 $$ f(t)=\sum_{m=0}^{n}S_{\alpha,m}(t-a) \bigl({}^{\mathrm{ABC}}_{}D_{a+}^{\alpha } \bigr)^{m}f(a)+S_{\alpha,n+1}(t-a) \bigl({}^{\mathrm{ABC}}_{}D_{a+}^{\alpha } \bigr)^{n+1}f(\xi) $$

*for some* $\xi\in(a,t)$, *where the function* *S* *is defined by*

7 $$ S_{\alpha,m}(x):=\sum_{k=0}^{m} \frac{\binom{m}{k}(1-\alpha )^{m-k}\alpha^{k}}{B(\alpha)^{m}\Gamma(k\alpha+1)}x^{k\alpha}. $$

#### Proof

The result of Lemma 2.1 can be rewritten as

$$\begin{aligned}& \bigl({}^{\mathrm{AB}}_{}I_{a+}^{\alpha} \bigr)^{m} \bigl({}^{\mathrm{ABC}}_{}D_{a+}^{\alpha} \bigr)^{m}f(t)- \bigl({}^{\mathrm{AB}}_{}I_{a+}^{\alpha } \bigr)^{m+1} \bigl({}^{\mathrm{ABC}}_{}D_{a+}^{\alpha} \bigr)^{m+1}f(t) \\& \quad =S_{\alpha,m}(t-a) \bigl({}^{\mathrm{ABC}}_{}D_{a+}^{\alpha} \bigr)^{m}f(a), \end{aligned}$$

valid for any $m\in\mathbb{N}$. Summing this identity over *m* to form a telescoping series, we get

$$f(t)- \bigl({}^{\mathrm{AB}}_{}I_{a+}^{\alpha} \bigr)^{n+1} \bigl({}^{\mathrm{ABC}}_{}D_{a+}^{\alpha} \bigr)^{n+1}f(t)=\sum_{m=0}^{n}S_{\alpha ,m}(t-a) \bigl({}^{\mathrm{ABC}}_{}D_{a+}^{\alpha} \bigr)^{m}f(a). $$

Thus it will suffice to prove that

8 $$ \bigl({}^{\mathrm{AB}}_{}I_{a+}^{\alpha} \bigr)^{n+1} \bigl({}^{\mathrm{ABC}}_{}D_{a+}^{\alpha} \bigr)^{n+1}f(t)=S_{\alpha,n+1}(t-a) \bigl({}^{\mathrm{ABC}}_{}D_{a+}^{\alpha} \bigr)^{n+1}f(\xi). $$

To establish (8), we use the mean value theorem for integrals once again, this time with one of the ‘functions’ involved being actually a distribution written in terms of the Dirac delta.

$$\begin{aligned} & \bigl({}^{\mathrm{AB}}_{}I_{a+}^{\alpha} \bigr)^{n+1} \bigl({}^{\mathrm{ABC}}_{}D_{a+}^{\alpha} \bigr)^{n+1}f(t) \\ &\quad = \biggl(\frac{1-\alpha}{B(\alpha)}+\frac{\alpha}{B(\alpha)}{}^{\mathrm{RL}}_{}I_{a+}^{\alpha} \biggr)^{n+1} \bigl({}^{ \mathrm{ABC}}_{}D_{a+}^{\alpha } \bigr)^{n+1}f(t) \\ &\quad =\sum_{k=0}^{n+1}\frac{\binom{n+1}{k}(1-\alpha)^{n+1-k}\alpha ^{k}}{B(\alpha)^{n+1}} {}^{\mathrm{RL}}_{}I_{a+}^{k\alpha} \bigl( \bigl({}^{ \mathrm{ABC}}_{}D_{a+}^{\alpha} \bigr)^{n+1}f(t) \bigr) \\ &\quad = \biggl(\frac{1-\alpha}{B(\alpha)} \biggr)^{n+1} \bigl({}^{ \mathrm{ABC}}_{}D_{a+}^{\alpha} \bigr)^{n+1}f(t) \\ &\qquad {} +\sum_{k=1}^{n+1} \frac{\binom{n+1}{k}(1-\alpha )^{n+1-k}\alpha^{k}}{B(\alpha)^{n+1}\Gamma(k\alpha)} \int_{a}^{t}(t-x)^{k\alpha -1} \bigl({}^{ \mathrm{ABC}}_{}D_{a+}^{\alpha} \bigr)^{n+1}f(x)\, \mathrm{d}x \\ &\quad = \int_{a}^{t} \Biggl[ \biggl(\frac{1-\alpha}{B(\alpha)} \biggr)^{n+1}\delta (t-x) +\sum_{k=1}^{n+1} \frac{\binom{n+1}{k}(1-\alpha )^{n+1-k}\alpha^{k}}{B(\alpha)^{n+1}\Gamma(k\alpha)}(t-x)^{k\alpha-1} \Biggr] \bigl({}^{\mathrm{ABC}}_{}D_{a+}^{\alpha} \bigr)^{n+1}f(x)\,\mathrm{d}x \\ &\quad = \bigl({}^{\mathrm{ABC}}_{}D_{a+}^{\alpha} \bigr)^{n+1}f(\xi) \int_{a}^{t} \Biggl[ \biggl(\frac{1-\alpha}{B(\alpha)} \biggr)^{n+1}\delta(t-x) \\ &\qquad {} +\sum_{k=1}^{n+1} \frac{\binom{n+1}{k}(1-\alpha )^{n+1-k}\alpha^{k}}{B(\alpha)^{n+1}\Gamma(k\alpha)}(t-x)^{k\alpha-1} \Biggr]\,\mathrm{d}x \\ &\quad = \bigl({}^{\mathrm{ABC}}_{}D_{a+}^{\alpha} \bigr)^{n+1}f(\xi) \Biggl[ \biggl(\frac{1-\alpha}{B(\alpha)} \biggr)^{n+1}+ \sum_{k=1}^{n+1}\frac{\binom {n+1}{k}(1-\alpha)^{n+1-k}\alpha^{k}}{B(\alpha)^{n+1}\Gamma(k\alpha +1)}(t-a)^{k\alpha} \Biggr] \\ &\quad =S_{\alpha,n+1}(t-a) \bigl({}^{\mathrm{ABC}}_{}D_{a+}^{\alpha} \bigr)^{n+1}f(\xi), \end{aligned}$$

as required. □

In order to get an infinite Taylor series expansion for a given function $f(t)$, it suffices to impose the following convergence condition on the remainder term:

9 $$ S_{\alpha,n}(t-a) \bigl\Vert \bigl({}^{\mathrm{ABC}}_{}D_{a+}^{\alpha} \bigr)^{n}f \bigr\Vert \rightarrow0 \quad \text{ as }n\rightarrow\infty, $$

where the norm used is the uniform norm on $[a,t]$.

One disadvantage of Theorem 2.3 is that for many functions *f*, the ABC fractional derivative ${}^{\mathrm{ABC}}_{}D^{\alpha }_{a+}\,f(t)$ evaluated at the starting point $t=a$ is zero. We can see this by considering the definition: since the ABC derivative is given by an integral from *a* to *t*, it will evaluate to zero given certain conditions on the behaviour of $f(t)$ near $t=a$. Thus, we present the following generalisation of Theorem 2.3, inspired by the work of [36].

#### Theorem 2.4

(AB Taylor series—general case)

*If* $\alpha\in(0,1)$ *and* $n\in\mathbb{N}$ *and* $a< b$ *in* $\mathbb{R}$ *and* $f:[a,b]\rightarrow\mathbb{R}$ *is a differentiable function such that* $f'$ *and all functions of the form* $({}^{\mathrm{ABC}}_{}D_{a+}^{\alpha} )^{m}f(t)$, $m\in\mathbb{N}$, *are* $L^{1}$ *functions*, *then for all* $c,t\in[a,b]$,

10 $$ f(t)=\sum_{m=0}^{n} \Delta_{m} \bigl({}^{\mathrm{ABC}}_{}D_{a+}^{\alpha} \bigr)^{m}f(c)+R_{n+1}, $$

*where the sequence of functions* $\Delta_{m}$ *is defined recursively by*

11 $$ \Delta_{0,k}=S_{\alpha,k}(t-a),\qquad \Delta_{m,k}=\Delta_{m-1,k}-\Delta _{m-1,m-1}S_{\alpha,k-m+1}(c-a) $$

*and* $\Delta_{m}=\Delta_{m,m}$, *the functions* $S_{\alpha,m}$ *being defined by* (7), *and the remainder term* $R_{n+1}$ *is a linear combination of terms of the form* $({}^{\mathrm{ABC}}_{}D_{a+}^{\alpha } )^{n+1}f(\xi)$ *for* $\xi\in(a,b)$.

#### Proof

We use formula (6) from Theorem 2.3 as our starting point, and apply it multiple times in different ways to derive (10).

Replacing *t* by *c* in equation (6), and replacing *f* by its ABC derivatives as appropriate, yields the following formulae for any fixed *n* (where we use the fact that $S_{\alpha,0}=1$):

$$\begin{aligned}& f(a) =f(c)-\sum_{m=1}^{n}S_{\alpha,m}(c-a) \bigl({}^{\mathrm{ABC}}_{}D_{a+}^{\alpha} \bigr)^{m}f(a) \\& \hphantom{f(a) ={}}{}-S_{\alpha ,n+1}(c-a) \bigl({}^{\mathrm{ABC}}_{}D_{a+}^{\alpha} \bigr)^{n+1}f(\xi_{0}), \\& {}^{\mathrm{ABC}}_{}D_{a+}^{\alpha}\,f(a) ={}^{ \mathrm{ABC}}_{}D_{a+}^{\alpha }\,f(c)-\sum _{m=1}^{n-1}S_{\alpha,m}(c-a) \bigl({}^{ \mathrm{ABC}}_{}D_{a+}^{\alpha} \bigr)^{m+1}f(a) \\& \hphantom{{}^{\mathrm{ABC}}_{}D_{a+}^{\alpha}\,f(a) ={}}{}-S_{\alpha,n}(c-a) \bigl({}^{\mathrm{ABC}}_{}D_{a+}^{\alpha } \bigr)^{n+1}f(\xi_{1}), \\& \bigl({}^{\mathrm{ABC}}_{}D_{a+}^{\alpha} \bigr)^{2}f(a) = \bigl({}^{\mathrm{ABC}}_{}D_{a+}^{\alpha} \bigr)^{2}f(c)-\sum_{m=0}^{n-2}S_{\alpha ,m}(c-a) \bigl({}^{\mathrm{ABC}}_{}D_{a+}^{\alpha} \bigr)^{m+2}f(a) \\& \hphantom{\bigl({}^{\mathrm{ABC}}_{}D_{a+}^{\alpha} \bigr)^{2}f(a) ={}}{}-S_{\alpha,n-1}(c-a) \bigl({}^{\mathrm{ABC}}_{}D_{a+}^{\alpha } \bigr)^{n+1}f(\xi_{2}), \\& \vdots \end{aligned}$$

Substituting each of these equations in turn into (6) yields the following sequence of identities:

$$\begin{aligned} f(t) =&\Delta_{0,0}f(a)+\sum_{m=1}^{n} \Delta_{0,m} \bigl({}^{\mathrm{ABC}}_{}D_{a+}^{\alpha} \bigr)^{m}f(a)+R_{0,n+1} \\ =&\Delta_{0,0}f(c)+\sum_{m=1}^{n} \bigl[\Delta_{0,m}-\Delta_{0,0}S_{\alpha ,m}(c-a) \bigr] \bigl({}^{\mathrm{ABC}}_{}D_{a+}^{\alpha} \bigr)^{m}f(a)+R_{1,n+1} \\ =&\Delta_{0,0}f(c)+\Delta_{1,1}{}^{\mathrm{ABC}}_{}D_{a+}^{\alpha}\,f(a)+\sum_{m=2}^{n} \Delta_{1,m} \bigl({}^{\mathrm{ABC}}_{}D_{a+}^{\alpha} \bigr)^{m}f(a)+R_{1,n+1} \\ =&\Delta_{0,0}f(c)+\Delta_{1,1}{}^{\mathrm{ABC}}_{}D_{a+}^{\alpha}\,f(c) \\ &{} +\sum_{m=2}^{n} \bigl[ \Delta_{1,m}-\Delta_{1,1}S_{\alpha ,m+1}(c-a) \bigr] \bigl( {}^{\mathrm{ABC}}_{}D_{a+}^{\alpha} \bigr)^{m}f(a)+R_{2,n+1} \\ =&\Delta_{0,0}f(c)+\Delta_{1,1}{}^{\mathrm{ABC}}_{}D_{a+}^{\alpha }\,f(c)+\Delta_{2,2} \bigl({}^{ \mathrm{ABC}}_{}D_{a+}^{\alpha} \bigr)^{2}f(a) \\ &{} +\sum_{m=3}^{n}\Delta_{2,m} \bigl({}^{\mathrm{ABC}}_{}D_{a+}^{\alpha} \bigr)^{m}f(a)+R_{2,n+1} \\ =&\cdots, \end{aligned}$$

where the $\Delta_{k,m}$ are defined by (11) and the successive remainders are given by

$$\begin{aligned}& R_{0,n+1} =\Delta_{0,n+1} \bigl({}^{\mathrm{ABC}}_{}D_{a+}^{\alpha} \bigr)^{n+1}f(\xi); \\& R_{k+1,n+1} =R_{k,n+1}-\Delta_{k,k}S_{\alpha,n-k+1}(c-a) \bigl({}^{\mathrm{ABC}}_{}D_{a+}^{\alpha} \bigr)^{n+1}f(\xi_{k}). \end{aligned}$$

After *n* iterations of this process, we arrive at the final result:

$$\begin{aligned} f(t) =&\Delta_{0,0}f(c)+\Delta_{1,1}{}^{\mathrm{ABC}}_{}D_{a+}^{\alpha }\,f(c)+\Delta_{2,2} \bigl({}^{ \mathrm{ABC}}_{}D_{a+}^{\alpha} \bigr)^{2}f(a) \\ &{}+\cdots+\Delta_{n,n} \bigl({}^{\mathrm{ABC}}_{}D_{a+}^{\alpha} \bigr)^{n}f(a)+R_{n,n+1}. \end{aligned}$$

Since $\Delta_{m}=\Delta_{m,m}$ by definition, and letting $R_{n+1}=R_{n,n+1}$, we discover equation (10) as required. Note that $\xi\in(a,t)$ and $\xi_{m}\in(a,c)$ for all *m*. □

Iterated ABC differintegrals to arbitrary order would be very difficult to compute directly. Fortunately, we can use the series formula from [26] to derive a significantly simpler expression for $({}^{\mathrm{ABC}}_{}D_{a+}^{\alpha} )^{m}f$ as follows:

12 $$\begin{aligned} \bigl({}^{\mathrm{ABC}}_{}D_{a+}^{\alpha} \bigr)^{m}f(t)&= \Biggl[\frac{B(\alpha)}{1-\alpha}\sum _{n=0}^{\infty} \biggl(\frac{-\alpha }{1-\alpha} \biggr)^{n}{}^{\mathrm{RL}}_{}I^{\alpha n+1}_{a+} \frac{\mathrm {d}}{\mathrm{d}t} \Biggr]^{m}f(t) \\ &=\frac{B(\alpha)^{m}}{(1-\alpha)^{m}}\sum_{n_{1},\ldots,n_{m}} \biggl( \frac{-\alpha}{1-\alpha} \biggr)^{\sum n_{i}}{}^{\mathrm{RL}}_{}I^{\alpha\sum n_{i}+1}_{a+}\frac{\mathrm{d}}{\mathrm{d}t}f(t) \\ &=\frac{B(\alpha)^{m}}{(1-\alpha)^{m}}\sum_{N=0}^{\infty} \binom {N+m-1}{m-1} \biggl(\frac{-\alpha}{1-\alpha} \biggr)^{N}{}^{ \mathrm{RL}}_{}I^{\alpha N+1}_{a+}\,f'(t), \end{aligned}$$

where this series is locally uniformly convergent in *t*. Using formula (12) for the iterated ABC derivative makes the Taylor series (6) and (10) easier to compute for specific individual functions *f*. See the next section for an example.

Unfortunately, given the complexity of the formula for the remainder term $R_{n+1}$, it will be difficult to tell whether and when series (10) converges as *n* goes to infinity. But we certainly have a valid finite series result, which can be verified computationally even for large values of *n*.

## Examples and applications

As a basic example of the main result Theorem 2.4, let us consider what the series looks like with the particular function $f(t)=(t-a)^{\beta}$.

Using expression (12) for the iterated ABC derivative, we find that in this case

13 $$\begin{aligned}& \bigl({}^{\mathrm{ABC}}_{}D_{a+}^{\alpha} \bigr)^{m}f(t) \\& \quad = \biggl(\frac {B(\alpha)}{1-\alpha} \biggr)^{m}\sum _{N=0}^{\infty}\binom{N+m-1}{m-1} \biggl(\frac{-\alpha}{1-\alpha} \biggr)^{N}\frac{\Gamma(\beta+1)}{\Gamma(\beta +\alpha N+1)}(t-a)^{\beta+\alpha N}. \end{aligned}$$

So the ABC Taylor series for this $f(t)$ is given by (10) with the iterated ABC derivatives and the coefficients $\Delta_{m}$ given respectively by (13) and (11). I.e.:

14 $$\begin{aligned} f(t) =&\sum_{m=0}^{n}\Delta_{m} \biggl(\frac{B(\alpha)}{1-\alpha} \biggr)^{m}\sum_{N=0}^{\infty} \binom{N+m-1}{m-1} \biggl(\frac{-\alpha}{1-\alpha} \biggr)^{N} \frac{\Gamma(\beta+1)}{\Gamma(\beta+\alpha N+1)}(c-a)^{\beta +\alpha N} \\ &{}+ \Biggl[\Delta_{0,n+1} \bigl({}^{\mathrm{ABC}}_{}D_{a+}^{\alpha } \bigr)^{n+1}(\xi-a)^{\beta} \\ &{}-\sum_{k=0}^{n-1}\Delta_{k,k}S_{\alpha ,n-k+1}(c-a) \bigl({}^{\mathrm{ABC}}_{}D_{a+}^{\alpha} \bigr)^{n+1}(\xi _{k}-a)^{\beta} \Biggr], \end{aligned}$$

where the Δ and *S* functions are defined by (11) and (7), and the constants $\xi,\xi_{1},\ldots,\xi_{n-1}$ are in the interval $(a,\max(c,t))$.

Finally, we shall present an application of the new Taylor series given by Theorem 2.3.

The paper [37] used a fractional Taylor series for Caputo derivatives, namely the result of [31], to derive a new fractional Boussinesq equation, assuming a power law for the changes of flux in a control volume, as well as deriving a linear form of the same equation under an extra physical assumption. In the paper [38], this differential equation was used to model a water table profile between two parallel subsurface drains in both homogeneous and heterogeneous soils, and this application was verified by experiment.

In the problem of modelling unconfined groundwater, the inflow component in the *x* and *y* directions of fluid mass flux is given by

15 $$\begin{aligned}& M(x) =\Delta y\rho q_{x}, \end{aligned}$$

16 $$\begin{aligned}& M(y) =\Delta x\rho q_{y}, \end{aligned}$$

where *ρ* is the fluid density and $q_{x}$, $q_{y}$ are the components in the *x* and *y* directions of the specific discharge. We assume that the change of flux in the *x* and *y* directions are power-law functions of order *α* and *β*, respectively. The fractional Taylor series for *M* given by (6) can be truncated after two terms to yield

17 $$\begin{aligned}& M(x+\Delta x) =\Delta y \biggl(\rho q_{x}+ \biggl[\frac{1-\alpha}{B(\alpha )}+ \frac{\alpha(\Delta x)^{\alpha}}{B(\alpha)\Gamma(\alpha+1)} \biggr]\frac{\partial^{\alpha}(\rho q_{x})}{\partial x^{\alpha}} \biggr), \end{aligned}$$

18 $$\begin{aligned}& M(y+\Delta y) =\Delta x \biggl(\rho q_{y}+ \biggl[\frac{1-\beta}{B(\beta )}+ \frac{\beta(\Delta y)^{\beta}}{B(\beta)\Gamma(\beta+1)} \biggr]\frac {\partial^{\beta}(\rho q_{y})}{\partial y^{\beta}} \biggr), \end{aligned}$$

where the *α*th derivatives here are defined by the ABC formula. Thus, subtracting equations (17)–(18) from equations (15)–(16), we get

19 $$\begin{aligned}& M(x)-M(x+\Delta x) =-\Delta y \biggl[\frac{1-\alpha}{B(\alpha)}+\frac {(\Delta x)^{\alpha}}{B(\alpha)\Gamma(\alpha)} \biggr]\frac{\partial ^{\alpha}(\rho q_{x})}{\partial x^{\alpha}}, \end{aligned}$$

20 $$\begin{aligned}& M(y)-M(y+\Delta y) =-\Delta x \biggl[\frac{1-\beta}{B(\beta)}+\frac {(\Delta y)^{\beta}}{B(\beta)\Gamma(\beta)} \biggr]\frac{\partial^{\beta }(\rho q_{y})}{\partial y^{\beta}}. \end{aligned}$$

The relevant equation describing water mass conservation is [39]

$$\begin{aligned}& \Delta t \bigl( \bigl[M(x)-M(x+\Delta x) \bigr]+ \bigl[M(y)-M(y+\Delta y) \bigr]+\rho N \bigr) \\& \quad =\rho\Delta x\Delta yS \bigl[z(t+\Delta t-z(t) \bigr], \end{aligned}$$

where *x*, *y*, *z* are the three dimensions. As $\Delta t\rightarrow0$, this becomes

$$\bigl[M(x)-M(x+\Delta x) \bigr]+ \bigl[M(y)-M(y+\Delta y) \bigr]+\rho N=\rho \Delta x\Delta yS\frac{\partial h}{\partial t}. $$

Substituting in equations (19)–(20) to this, we find the following equation:

21 $$\begin{aligned}& {-} \biggl[(\Delta x)^{-1}\frac{1-\alpha}{B(\alpha)}+\frac{(\Delta x)^{\alpha-1}}{B(\alpha)\Gamma(\alpha)} \biggr] \frac{\partial^{\alpha }(\rho q_{x})}{\partial x^{\alpha}} \\& \quad {}- \biggl[(\Delta y)^{-1}\frac{1-\beta }{B(\beta)}+\frac{(\Delta y)^{\beta-1}}{B(\beta)\Gamma(\beta)} \biggr]\frac{\partial^{\beta}(\rho q_{y})}{\partial y^{\beta}}+\rho N=\rho S\frac{\partial h}{\partial t}. \end{aligned}$$

Assuming that *ρ* is constant (i.e. that the fluid is incompressible), equation (21) becomes

22 $$\begin{aligned} \begin{aligned}[b] &{-} \biggl[(\Delta x)^{-1}\frac{1-\alpha}{B(\alpha)}+\frac{(\Delta x)^{\alpha-1}}{B(\alpha)\Gamma(\alpha)} \biggr] \frac{\partial^{\alpha }q_{x}}{\partial x^{\alpha}} \\ &\quad {}- \biggl[(\Delta y)^{-1}\frac{1-\beta }{B(\beta)}+\frac{(\Delta y)^{\beta-1}}{B(\beta)\Gamma(\beta)} \biggr]\frac{\partial^{\beta}q_{y}}{\partial y^{\beta}}+N=S\frac{\partial h}{\partial t}. \end{aligned} \end{aligned}$$

Thus we obtain a fractional partial differential equation of Boussinesq type to model unconfined groundwater. This differs from the other equations suggested so far in the literature, because of the Mittag–Leffler kernel used to define the fractional derivative.

## Conclusions

During the last few years, a lot of attention was paid to modelling the dynamics of anomalous systems using fractional calculus. In our view, the best way is to start with fundamental principles appearing in nature, and after that to apply fractional techniques.

In this manuscript, we have proved the mean value theorem and Taylor’s theorem for derivatives defined in terms of a Mittag–Leffler kernel. Formulae (6) and (10) obtained for Taylor’s theorem in the ABC context appear different from classical and previous results, mainly due to the replacement of power functions with a more general form of summand.

These results can be used to model real-world problems such as the motion of unconfined groundwater, and we hope that they may find more such applications in the future.

## References

[1] Caputo M. (1967). Linear models of dissipation whose *Q* is almost frequency independent—II. Geophys. J. Int..

[2] Kilbas A.A., Srivastava H.M., Trujillo J.J. (2006). Theory and Applications of Fractional Differential Equations.

[3] Podlubny I. (1999). Fractional Differential Equations.

[4] Samko S.G., Kilbas A.A., Marichev O.I. (2002). Fractional Integrals and Derivatives: Theory and Applications.

[5] Hilfer R. (2000). Applications of Fractional Calculus in Physics.

[6] Caputo M., Fabrizio M. (2015). A new definition of fractional derivative without singular kernel. Prog. Fract. Differ. Appl..

[7] Atangana A., Baleanu D. (2016). New fractional derivatives with nonlocal and non-singular kernel: theory and application to heat transfer model. Therm. Sci..

[8] Katugampola U.N. (2014). A new approach to generalized fractional derivatives. Bull. Math. Anal. Appl..

[9] Luchko Y., Yamamoto M. (2016). General time-fractional diffusion equation: some uniqueness and existence results for the initial-boundary-value problems. Fract. Calc. Appl. Anal..

[10] Fallahgoul H., Focardi S., Fabozzi F. (2016). Fractional Calculus and Fractional Processes with Applications to Financial Economics: Theory and Application.

[11] Atangana A. (2017). Fractional Operators with Constant and Variable Order with Application to Geo-Hydrology.

[12] Petras I. (2011). Fractional-Order Nonlinear Systems: Modeling, Analysis and Simulation.

[13] Carvalho A.R.M., Pinto C.M.A., Baleanu D. (2018). HIV/HCV coinfection model: a fractional-order perspective for the effect of the HIV viral load. Adv. Differ. Equ..

[14] Pinto C.M.A., Carvalho A.R.M. (2017). The HIV/TB coinfection severity in the presence of TB multi-drug resistant strains. Ecol. Complex..

[15] Pinto, C.M.A., Carvalho, A.R.M.: The impact of pre-exposure prophylaxis (PrEP) and screening on the dynamics of HIV. J. Comput. Appl. Math. (2017, in press). 10.1016/j.cam.2017.10.019

[16] Dokoumetzidis A., Macheras P. (2009). Fractional kinetics in drug absorption and disposition processes. J. Pharmacokinet. Pharmacodyn..

[17] Petráš I., Magin R.L. (2011). Simulation of drug uptake in a two compartmental fractional model for a biological system. Commun. Nonlinear Sci. Numer. Simul..

[18] Popović J.K., Spasić D.T., Tošić J., Kolarović J.L., Malti R., Mitić I.M., Pilipović S., Atanacković T.M. (2015). Fractional model for pharmacokinetics of high dose methotrexate in children with acute lymphoblastic leukaemia. Commun. Nonlinear Sci. Numer. Simul..

[19] Sopasakis P., Sarimveis H., Macheras P., Dokoumetzidis A. (2018). Fractional calculus in pharmacokinetics. J. Pharmacokinet. Pharmacodyn..

[20] Ray S.S. (2015). Fractional Calculus with Applications for Nuclear Reactor Dynamics.

[21] Mainardi F. (2010). Fractional Calculus and Waves in Linear Viscoelasticity.

[22] West B.J. (2015). Fractional Calculus View of Complexity: Tomorrow’s Science.

[23] Magin R.L. (2006). Fractional Calculus in Bioengineering.

[24] Ostalczyk P. (2016). Discrete Fractional Calculus: Applications in Control and Image Processing.

[25] Baleanu D., Diethelm K., Scalas E., Trujillo J.J. (2012). Fractional Calculus: Models and Numerical Methods.

[26] Baleanu D., Fernandez A. (2018). On some new properties of fractional derivatives with Mittag–Leffler kernel. Commun. Nonlinear Sci. Numer. Simul..

[27] Abdeljawad T., Baleanu D. (2017). Integration by parts and its applications of a new nonlocal fractional derivative with Mittag–Leffler nonsingular kernel. J. Nonlinear Sci. Appl..

[28] Alkahtani B.S.T. (2016). Chua’s circuit model with Atangana–Baleanu derivative with fractional order. Chaos Solitons Fractals.

[29] Gómez-Aguilar J.F. (2017). Irving–Mullineux oscillator via fractional derivatives with Mittag–Leffler kernel. Chaos Solitons Fractals.

[30] Trujillo J.J., Rivero M., Bonilla B. (1999). On a Riemann–Liouville generalized Taylor’s formula. J. Math. Anal. Appl..

[31] Odibat Z.M., Shawagfeh N.T. (2007). Generalized Taylor’s formula. Appl. Math. Comput..

[32] Abdeljawad T., Baleanu D. (2017). Monotonicity results for fractional difference operators with discrete exponential kernels. Adv. Differ. Equ..

[33] Abdeljawad T., Baleanu D. (2017). Monotonicity analysis of a nabla discrete fractional operator with discrete Mittag–Leffler kernel. Chaos Solitons Fractals.

[34] Djida J.D., Atangana A., Area I. (2017). Numerical computation of a fractional derivative with non-local and non-singular kernel. Math. Model. Nat. Phenom..

[35] Pollard H. (1948). The completely monotonic character of the Mittag–Leffler function $E_{\alpha}(-x)$. Bull. Am. Math. Soc..

[36] Usero, D.: Fractional Taylor Series for Caputo Fractional Derivatives. Construction of Numerical Schemes. Preprint http://www.fdi.ucm.es/profesor/lvazquez/calcfrac/docs/paper_Usero.pdf (2008). Accessed March 2018

[37] Mehdinejadiani B., Jafari H., Baleanu D. (2013). Derivation of a fractional Boussinesq equation for modelling unconfined groundwater. Eur. Phys. J. Spec. Top..

[38] Mehdinejadiani B., Naseri A.A., Jafari H., Ghanbarzadeh A., Baleanu D. (2013). A mathematical model for simulation of a water table profile between two parallel subsurface drains using fractional derivatives. Comput. Math. Appl..

[39] Bear J. (1972). Dynamics of Fluids in Porous.

